# Alfred Sheen

**Published:** 1907-03

**Authors:** 


### ALFRED SHEEN, M.D. St. Andrews, M.R.C.S. England, D.P.H. Cantab.

BORN at Leicester in 1839, and educated at Hurstpierpoint, Alfred Sheen found the routine of a Civil Service appointment at Madras uncongenial, and in 1856 he entered the Madras Medical College, where he carried off many prizes, and found time to be a leading member of the Madras Cathedral choir. Yet unqualified, he returned to England in medical charge of a large ship. His student days were completed at Guy's Hospital, where he obtained the Physical Society's prize for an essay on "Dysentery." In 1862 he qualified. In the following year the "Guyite Club" of 1863 was founded, of which he remained a member to the time of his death. Alfred Sheen's life work in Cardiff began in 1864, when he was appointed House Surgeon to the Cardiff Infirmary, at that time a small institution of some thirty beds. The House Surgeon was not only in charge inside, but also had to visit sick in the town, patients of the charity, and act as Secretary and General Superintendent. In this, his earliest work in Cardiff, Alfred Sheen acquitted himself well, displaying that abundant energy and power of work which were so characteristic of all his after life. He it was who first organised the "Infirmary Balls," ever since a successful annual function. In 1866 private practice was commenced; marriage and appointment to the Infirmary Honorary Staff followed shortly afterwards. Active practice was continued for forty years; indeed, to within a month of his death.

During his many years as Surgeon to the Cardiff Infirmary Alfred Sheen showed the greatest zeal, thoroughness and industry in his surgical work. He gave much time to the institution, and in the earlier days, when actual operations were not so numerous, much of the "case-taking" and dressing was done by his own hands. His perfect ambidexterity was a feature of his operating. The writer recalls best his transparent pleasure at the rapid and successful completion of an ovariotomy, and the slow, painstaking and methodical way in which he would dissect out a mass of tuberculous glands from the neck. Naturally at a time of change numerous innovations and additions were due to him; *inter alia*, he was the first to displace "antiseptic" by "aseptic" methods in Cardiff. In the numerous general duties which fall to a hospital administrator Alfred Sheen was always to the fore. When the Infirmary was moved to its present position in 1883, it was in the main due to his efforts that the site was obtained from the Marquis of Bute. He superintended every detail of its building, and, in addition, was an indefatigable collector of money to start it free from the burden of debt. It would not be too much to say that the Cardiff Infirmary building as it now exists stands as a monument to Alfred Sheen.

Space forbids reference to Alfred Sheen's many other activities—Initiator, Secretary and Treasurer of the South Wales branch, and until recently member of the Central Council, of the British Medical Association; Visiting Medical Officer to the Cardiff Workhouse Hospital, and an authority on Poor Law Medical Work; an original member of the Cardiff Medical Society, its first Secretary, at one time its President, and its first Honorary Member; Initiator, Treasurer, Chairman of Committee and Honorary Medical Officer of the Cardiff branch of the "Jubilee

Nurses" ; a most active and useful member of the Council of University College, Cardiff ; Treasurer and Hon. Surgeon-Colonel of the Church Lads' Brigade ; Churchwarden of St. German's, Cardiff, to the time of his death—these are some of the many active and useful ways in which his life was spent.

There are certain members of the community to whom people instinctively go in time of trouble, and Alfred Sheen was one of these. All through his life, by correspondence or by interview, there came to him many who needed advice and help on various subjects, and advice and help were always willingly, carefully, and thoroughly given. Underneath a reticent and somewhat blunt manner was concealed an exceeding kindness of heart. His medical publications were as follows :—

BIBLIOGRAPHY OF ALFRED SHEEN.

"Strangulated Inguinal Hernia ; operation ; death ; necropsy."—*Lancet*, 1878, ii. 879.
"Poisoning by Phosphorus ; post-mortem examination."—*Brit. M. J.*, 1879, i. 347.
"On Iodoform."—*Practitioner*, 1879, xxii. 321—324.
"Intestinal Obstruction ; puncture of the intestine ; left lumbar colotomy ; recovery."—*Brit. M. J.*, 1879, ii. 733.
"Provident Dispensaries."—*Ibid.*, 1880, i. 753.
"Five Years' Surgical Work in the Cardiff Infirmary."—*Lancet*, 1880, ii. 532, 613, 688, 764, 928.
"Colotomy in the Left Loin."—*Ibid.*, 929.
"Can a threatened attack of Diphtheria be averted."—*Ibid.*, 1881, ii. 1150.
"Two cases of Aneurysm of the Femoral Artery with Ligature of the External Iliac Artery ; recovery."—*Brit. M. J.*, 1882, ii. 720.
"Nitrite of Amyl and Nitro-Glycerine in Uræmic Asthma."—*Ibid.*, 1883, i. 811.
"An Epidemic of Tetanus."—*Bristol M.-Chir. J.*, 1883, i. 173—183.
"Three cases of Intestinal Obstruction."—*Ibid.*, 1884, ii. 167—174 ; also *Brit. M. J.*, 1884, ii. 1014.
"An Address on the Relations of the Medical Profession."—*Brit. M. J.*, 1884, ii. 896—901.
"On Diphtheria, with cases."—*Bristol M.-Chir. J.*, 1885, iii. 113—124.
"Strangulated Hernia and its Treatment."—*Brit. M. J.*, 1887, i. 387—389.
"Case of Stricture of Æsophagus ; gastrostomy ; death."—*Ibid.*, 1889, i. 1463.
"Two cases of Trephining."—*Ibid.*, 1890, i. 236—238.
"Pleural Effusions, their Diagnosis and Treatment."—*Bristol M.-Chir. J.*, 1893, xi. 14—29.
*The Workhouse and its Medical Officer.* 2nd edition. Bristol : Wright & Co. 1890.
"Selections from my Casebooks."—*Brit. M. J.*, 1895, i. 9—12.
"A case of Axial Rotation of the Testis."—*Lancet*, 1896, i. 990.
"On an Outbreak of Small-pox at the Workhouse, Cardiff."—*Brit. M. J.*, 1896, i. 835—837.
"Three Severe Operations in one Patient. (1) Abdominal Incision for Perityphlitic Abscess ; (2) Cœliotomy ; (3) Hepatomy for Abscess in Liver."—*Practitioner*, 1896, lvi. 607—611.
"A case of Puerperal Septicæmia treated by Antistreptococcic Serum ; death."—*Brit. M. J.*, 1896, ii. 1774.
"Case of Traumatic Rupture of the Duodenum and Jejunum."—*Ibid.*, 1899, i. 470.
"Recovery from Morphine Poisoning after Subcutaneous Injection of Atropine."—*Ibid.*, 1905, i. 1040.

*Also papers on*
"Indoor Medical Relief."
"Women's Work in Populous Parishes."
"Hospital Accommodation for Infectious Diseases in Cardiff."

### Local Medical Notes.

